# Global Public Interests and Dynamic Trends in Osteoporosis From 2004 to 2019: Infodemiology Study

**DOI:** 10.2196/25422

**Published:** 2021-07-05

**Authors:** Peng Wang, Qing Xu, Rong-Rong Cao, Fei-Yan Deng, Shu-Feng Lei

**Affiliations:** 1 Center for Genetic Epidemiology and Genomics, School of Public Health Soochow University Medical College Soochow University Suzhou China

**Keywords:** global public interest, Google trends, osteoporosis, seasonality, trends, infodemiology, information seeking, web-based information

## Abstract

**Background:**

With the prolonging of human life expectancy and subsequent population aging, osteoporosis (OP) has become an important public health issue.

**Objective:**

This study aimed to understand the global public search interests and dynamic trends in “osteoporosis” using the data derived from Google Trends.

**Methods:**

An online search was performed using the term “osteoporosis” in Google Trends from January 1, 2004, to December 31, 2019, under the category “Health.” Cosinor analysis was used to test the seasonality of relative search volume (RSV) for “osteoporosis.” An analysis was conducted to investigate the public search topic rising in RSV for “osteoporosis.”

**Results:**

There was a descending trend of global RSV for “osteoporosis” from January 2004 to December 2014, and a slowly increasing trend from January 2015 to December 2019. Cosinor analysis showed significant seasonal variations in global RSV for “osteoporosis” (*P*=.01), with a peak in March and a trough in September. In addition, similar decreasing trends of RSV for “osteoporosis” were found in Australia, New Zealand, Ireland, and Canada from January 2004 to December 2019. Cosinor test revealed significant seasonal variations in RSV for “osteoporosis” in Australia, New Zealand, Canada, Ireland, UK, and USA (all *P*<.001). Furthermore, public search rising topics related to “osteoporosis” included denosumab, fracture risk assessment tool, bone density, osteopenia, osteoarthritis, and risk factor.

**Conclusions:**

Our study provided evidence about the public search interest and dynamic trends in OP using web-based data, which would be helpful for public health and policy making.

## Introduction

### Overview

Osteoporosis (OP) is a generalized skeletal disorder characterized by reduction in bone mineral content, low bone mineral density, and deterioration of bone structure, all of which will eventually lead to bone fragility and an increased susceptibility to fracture [1-3]. OP is commonly described as a “silent disease” because it lacks obvious signs, and has now become an important public health issue, with estimates indicating that nearly 200 million individuals are impacted worldwide [4]. Multiple risk factors are thought to contribute to the increased likelihood of OP, including sex, age, race, hormone levels, dietary factors, and lifestyle choices [5-7].

Globally, internet has become an important platform for people to seek and share health-related information. The vast amount of new data derived from social media and search engines has shown potential values in investigating, nowcasting, and forecasting human behaviors and diseases [8,9]. Research on internet data are usually referred to as “infodemiology studies” [10,11].

Infodemiology is defined as “the science of distribution and determinants of information in an electronic medium, specifically the Internet, or in a population, with the ultimate aim to inform public health and public policy” [10,11]. Infodemiology data can be collected from social media sources (Facebook, Twitter, and Instagram) and search engines (Google, Bing, and Baidu) in near real-time [12,13]. Such data allow to predict outbreaks of diseases, aid in monitoring disease syndromic surveillance as well as in detecting and quantifying disparities in health information availability [14].

### Google Trends

Google Trends, as an accessible online tool, provides both real-time and archived information on Google search queries from 2004. So far, many studies have demonstrated that Google Trends could be regarded as a reliable tool for examining human behaviors, measuring the change in interest in controversial issues, analyzing public’s reaction to various outbreaks or incidents, and investigating seasonal trends related to various diseases and health issues [15-20]. This new approach is also gaining importance in disease surveillance studies and could serve as an effective complement to traditional, time-consuming survey methods [21-25].

Google Trends has increasingly become a meaningful health source for both laypeople and health professionals. The web-based information on Google Trends has been recognized as a surrogate tool for estimating epidemiology and gathering data on patterns of disease and human behaviors [15,20,23]. In fact, data from internet sources could serve as a real-time surveillance tool and a supplement for health care systems, so as to allocate appropriate resources for specific moments with higher disease burden.

Google Trends, however, has not yet been used to investigate the public search interest and trends in OP. Therefore, we conducted this study to better understand the utility of Google Trends data for exploring global public search interest and dynamic trends in OP over time.

## Methods

### Keyword Selection

Data on internet search for “osteoporosis” were obtained from Google Trends, which provided the relative search volume (RSV) for the aforesaid search term. To make reasonable comparisons between different search terms, Google Trends adjusts the search results to the time and location of a search term by dividing each data point by the total searches of the geography and period, and then by scaling these resulting numbers based on a given search term’s proportion. The higher scores represent higher RSV. The data points can be downloaded from Google Trends in “.csv” format. To avoid selection bias, Google Trends excludes all of the repeated searches from the same person during a short span of time [15].

### Region and Period Selection

On April 20, 2020, the keyword “osteoporosis” was searched by individually selecting the countries “Worldwide,” “Australia,” “New Zealand,” “Canada,” “Ireland,” “UK,” and “USA” and choosing the category as “Health.” The corresponding time-series data from January 2004 to December 2019 were then downloaded. The time-series data in our study were not a product of comparison between countries, but rather a longitudinal data on the RSV for “osteoporosis” in a single country. The period selection was representative and appropriate for our study, and contained retrospective data over the past 16 years.

### Statistical Analysis

Cosinor analysis was utilized to investigate the seasonal patterns of RSV for “osteoporosis,” where the RSV was regressed onto a sine and a cosine term of transformations of the time variable and represented as a sine curve that could be applied to test the seasonality [26,27]. A time-series plot was used to demonstrate the consistency in seasonal patterns. Statistical analysis on seasonality was conducted using the “season” package in R version 3.4.4 (R Foundation for Statistical Computing), while an analysis on OP-related topics was performed using Google Trends data [28]. Statistical significance was set as 2-tailed *P*<.05.

### Availability of Data and Material

The data and material that support the findings of this study are available from public data sets that could be found in Google Trends.

## Results

### Global Search Popularity for “Osteoporosis”

**Figure 1:**
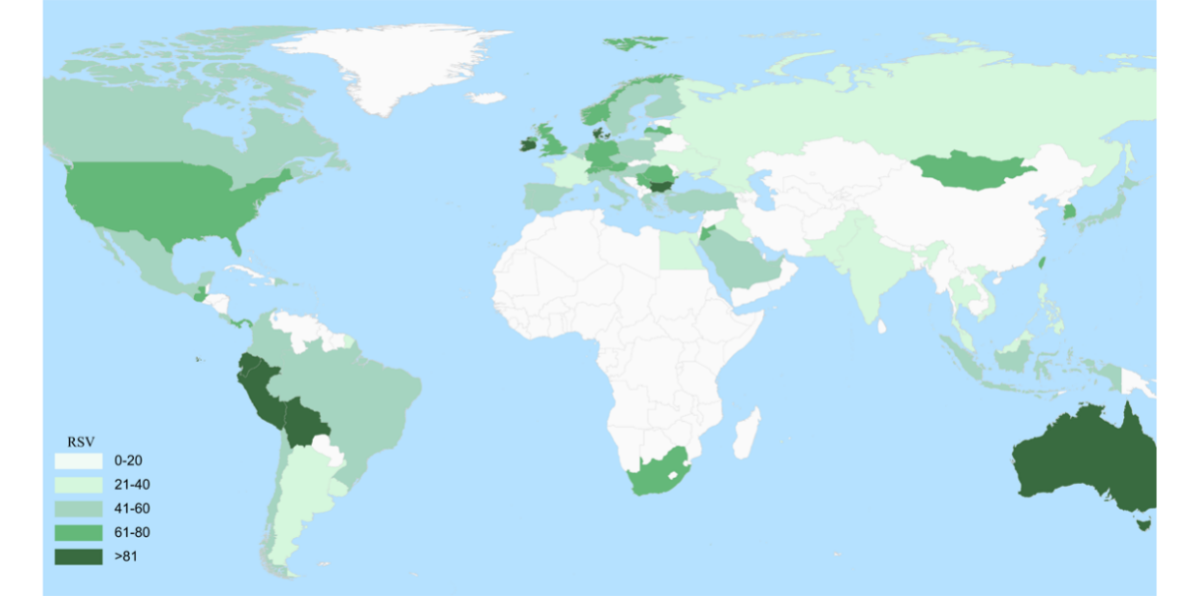
Graphic map of search popularity for “osteoporosis” by location (worldwide). RSV: relative search volume.

As displayed in Figure 1, the color intensity represents the RSV of Google searches performed during the studied period. The highest RSV for “osteoporosis” was found in Bolivia (100), Peru (95), Ireland (95), Australia (91), Bulgaria (87), Denmark (87), Singapore (73), Mongolia (73), South Africa (73), UK (69), New Zealand (69), USA (65), Panama (65), Puerto Rico (60), and Canada (60).

### Global Search Trend and Seasonal Patterns for RSV of “Osteoporosis”

**Figure 2:**
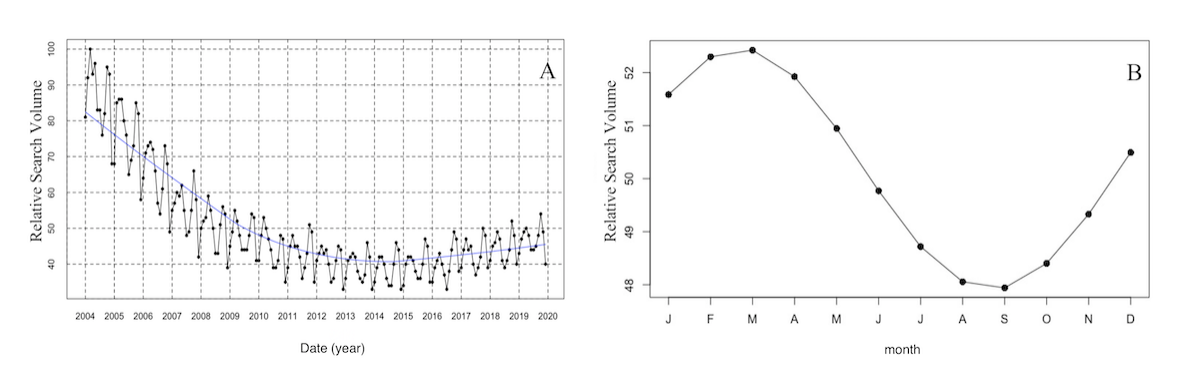
Time series plots for the worldwide relative search volume of osteoporosis from January 01, 2004, to December 31, 2019 (A), and the plots of cosinor models for the seasonal variation in the worldwide relative search volume of osteoporosis (B).

On a worldwide scale, there was a descending trend of RSV for “osteoporosis” from January 2004 to December 2014, and a slowly increasing trend from January 2015 to December 2019 (Figure 2A). Moreover, cosinor analysis suggested a significant seasonal pattern in RSV for “osteoporosis” (*P*=.01), with a peak in March and a trough in September (Figure 2B and Table 1).

**Table 1:** The seasonal variations in relative search volume for “osteoporosis.”

Location	Number of observations	Amplitude	Phase month	Low point month	*P* value^a^
Worldwide	192	2.3	2.7	8.7	.01
Australia	192	7.3	6.3	12.3	<.001
New Zealand	192	2.7	5.7	11.7	<.001
Canada	192	6.2	1.5	7.5	<.001
Ireland	192	3.6	2.0	8.0	<.001
UK	192	5.7	2.2	8.2	<.001
USA	192	4.2	1.7	7.7	<.001

^a^Statistical significance was set as *P* value <.05.

### Search Trend and Seasonal Pattern for RSV of “Osteoporosis” in Six English-Speaking Countries

**Figure 3:**
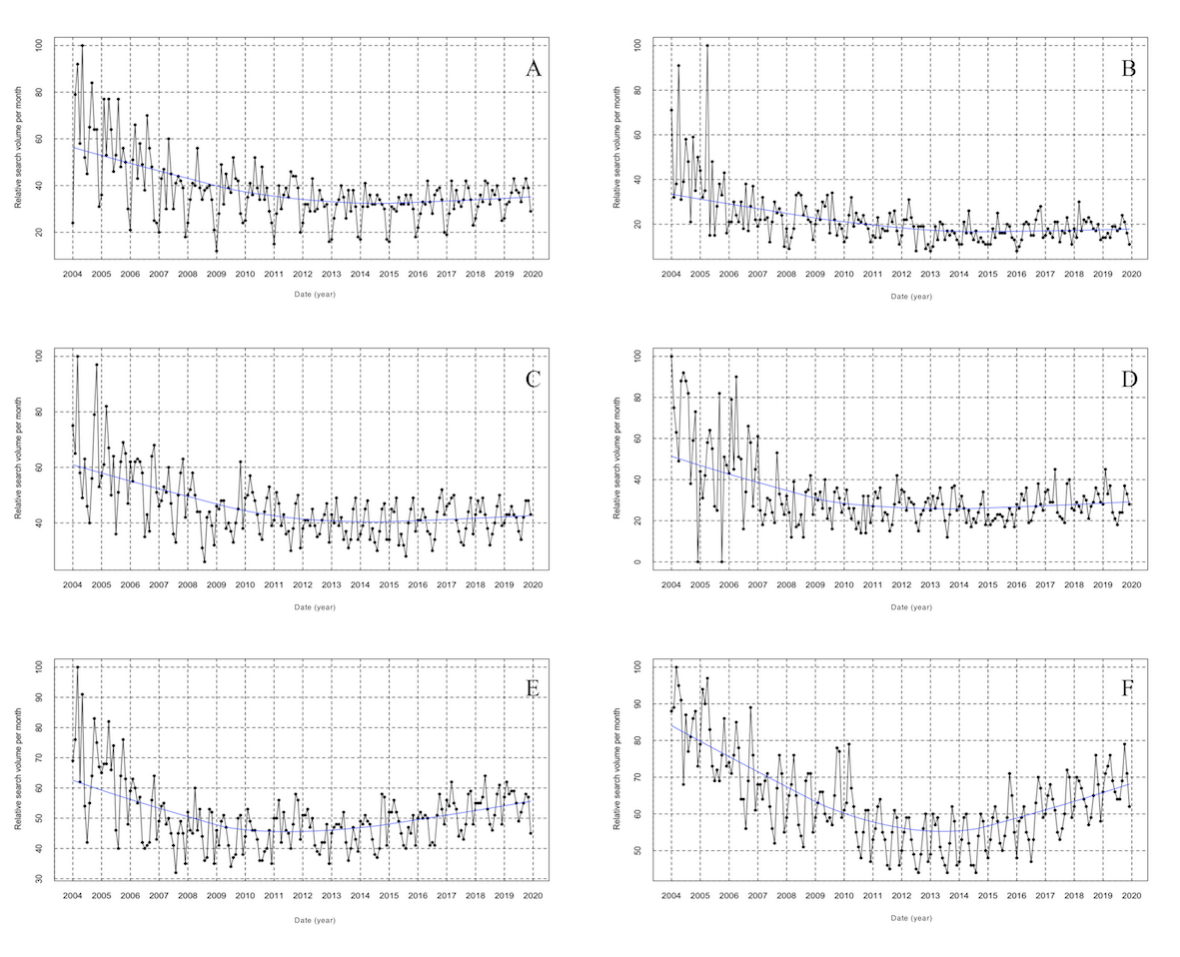
Time series plots for the relative search volume of osteoporosis Australia (A), New Zealand (B), Canada (C), Ireland (D), UK (E), and USA (F) from January 01, 2004, to December 31, 2019.

A similar decreasing trend of RSV for “osteoporosis” was found in Australia, New Zealand, Ireland, and Canada from January 2004 to December 2019 (Figure 3A-D). In the UK, there was first a decreasing trend (from January 2004 to December 2012) and then a progressively increasing trend (from January 2013 to December 2019) of RSV for “osteoporosis” (Figure 3E). In addition, RSV for “osteoporosis” in the USA presented a descending trend from January 2004 to December 2014, and an increasing trend from January 2015 to December 2019 (Figure 3F).

**Figure 4:**
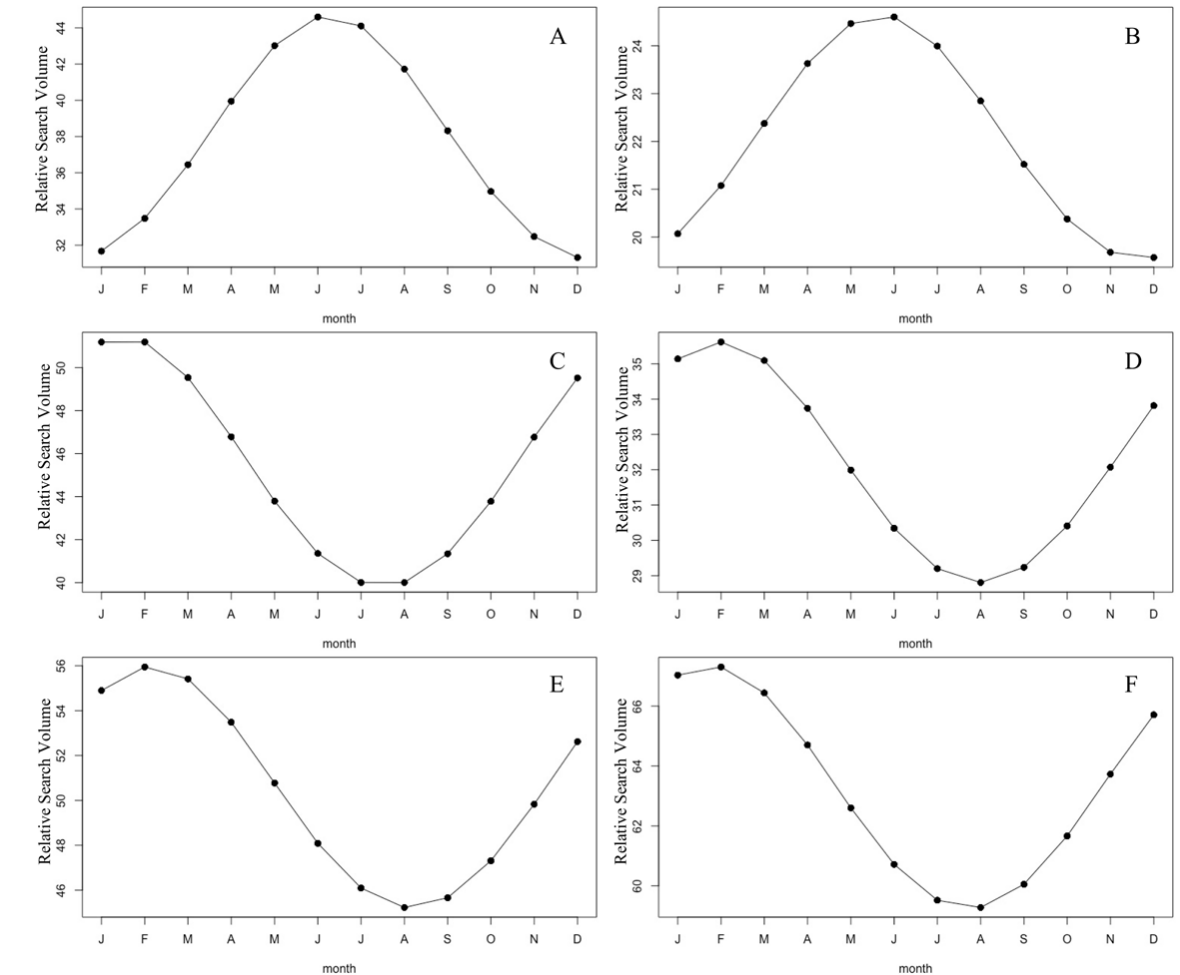
The plots of cosinor models for the seasonal variation in the relative search volume of osteoporosis in Australia (A), New Zealand (B), Canada (C), Ireland (D), UK (E), and USA (F) from January 01, 2004, to December 31, 2019.

The results of cosinor test showed significant seasonal variations of RSV for “osteoporosis” in Australia, New Zealand, Canada, Ireland, UK, and USA (all *P*<.001; Figure 4; Table 1). RSV for “osteoporosis” in 2 southern hemisphere countries (Australia and New Zealand) peaked in winter months (June/July) and was at its lowest point in summer months (December/January; Figure 4A and B), whereas in 4 northern hemisphere countries (Canada, Ireland, UK, and USA), RSV for “osteoporosis” showed a peak in winter months (January/February) and a nadir in late summer/early autumn months (July/August/September; Figure 4C-F). There was an approximate 6-month difference in RSV for “osteoporosis” between southern and northern hemisphere countries with a reversed meteorological month (Figure 4).

### Rise in Public Relative Search Topics Regarding “Osteoporosis”

The rise in relative search topics was compared with the last period. The results showed that the global top rising topics were denosumab, fracture risk assessment tool (FRAX), bone tumor, hip fracture, osteomalacia, bone density, risk factor, osteoarthritis, arthritis, and osteopenia (Table 2). As for the 6 English-speaking countries, we observed that the most search rising topics were bone, bone density, osteopenia, osteoarthritis, risk factor, preventive healthcare, and the medications used for OP treatment (including denosumab, teriparatide, bisphosphonate; Table 2).

**Table 2:** Public relative search topics rising in “osteoporosis.”

Rank	Worldwide	Australia	New Zealand	Canada	Ireland	UK	USA
	Search topics (% rising)	Search topics (% rising)	Search topics (% rising)	Search topics (% rising)	Search topics (% rising)	Search topics (% rising)	Search topics (% rising)
1	Denosumab (breakout^a^)	Bone (breakout)	Bone (breakout)	Osteoarthritis (breakout)	Bone (breakout)	Bone density (breakout)	Risk factor (breakout)
2	FRAX^b^ (breakout)	Calcium (breakout)	Osteoarthritis (breakout)	Guideline (breakout)	Osteoarthritis (breakout)	Osteopenia (breakout)	Denosumab (breakout)
3	Bone tumor (breakout)	Bone density (breakout)	Calcium (breakout)	Denosumab (breakout)	Irish Osteoporosis Society (breakout)	Dual-energy X-ray absorptiometry (breakout)	Teriparatide (breakout)
4	Hip fracture (breakout)	Preventive healthcare (breakout)	Vitamin D (breakout)	Bisphosphonate (breakout)	Calcium (breakout)	Osteomalacia (breakout)	Ibandronic acid (breakout)
5	Osteomalacia (1100)	Osteopenia (breakout)	Osteomalacia (breakout)	Risk factor (breakout)	Osteopenia (breakout)	FRAX (breakout)	Osteomyelitis (breakout)
6	Bone density (550)	Rheumatoid arthritis (breakout)	Bone density (breakout)	Risedronic acid (breakout)	Royal Osteoporosis Society (breakout)	Osteoarthritis (750)	Osteogenesis imperfecta (breakout)
7	Risk factor (450)	Risk factor (breakout)	Osteopenia (breakout)	Bone (650)	None	Calcium (300)	Mineral (breakout)
8	Osteoarthritis (400)	Denosumab (breakout)	Osteoporosis (40)	Osteoporosis (190)	None	Preventive healthcare (250)	Osteoarthritis (950)
9	Arthritis (250)	Osteoarthritis (300)	None	Preventive healthcare (180)	None	Osteoporosis (150)	Risk (450)
10	Osteopenia (140)	Osteoporosis (190)	None	Bone density (170)	None	Bone (60)	Bone (200)

^a^“Breakout” represents that the search term grew by more than 5000% compared with previous period.

^b^FRAX: fracture risk assessment tool.

## Discussion

### Principal Findings

In this study, we observed that the global internet search interest in “osteoporosis” steadily decreased from January 2004 to December 2014, whereas it slowly increased from January 2015 to December 2019. In addition, the presence of seasonal pattern in RSV for “osteoporosis” was revealed, with a peak in March and a trough in September. Moreover, we have investigated the RSV for “osteoporosis” among 6 English-speaking countries, which provided a good representation of countries in both northern and southern hemispheres. Similar change trends of RSV for “osteoporosis” were found for USA and UK as well; however, descending trends of RSV for “osteoporosis” were noted for Australia, New Zealand, Ireland, and Canada. Furthermore, the seasonal variations in RSV for “osteoporosis” among 6 English-speaking countries were confirmed, with a peak in late winter/early spring months and nadir in late summer/early autumn months. There was a nearly 6-month difference in the RSV for “osteoporosis” between southern/northern hemisphere countries with a reversed meteorological month, suggesting the presence of seasonal variations rather than calendar-driven patterns. The dynamic trends in “osteoporosis” could provide insights into the epidemiology of OP, as well as help care professionals and policy makers anticipate and prepare for this disease.

Rise in relative search topics on OP was also analyzed, with “denosumab” and “FRAX” identified as the top 2 global rising topics. Among 6 English-speaking countries, “bone” and “medications used for osteoporosis treatment” represented 2 of the most searched topics. Denosumab, also called receptor activator of nuclear factor-kappa B (RANK) ligand inhibitor, is a human monoclonal antibody used to increase bone mass and strength in the treatment of OP. The similar public search topic rising of “medications used for osteoporosis treatment” worldwide and in 6 English-speaking countries reflects the increasing public awareness of the treatment for OP other than the disease itself. FRAX is a diagnostic tool used to evaluate the probability of incurring an osteoporotic fracture. This topic rising may imply the public concerns about the possible osteoporotic fracture risk. The search topic rising interests in OP are of great importance for doctors and nurses, as they can capture the fluctuations of fast-growing topics of patients with OP and provide timely health promotion and education.

A number of studies have investigated the seasonal variation in OP presentation, and suggested that the seasonal variation in vitamin D concentration may be relevant to the occurrence of OP [29-32]. In a Greek cohort of 596 postmenopausal women with OP, there was a seasonal variation in serum levels of 25-hydroxy vitamin D [25(OH)D], with the highest and lowest 25(OH)D levels noted in late summer/early autumn months (August/September/October) and late winter/early spring months (March), respectively. Klenk et al [33] have also demonstrated a seasonal effect on the serum 25(OH)D levels in southern Germany, with the minimum 25(OH)D serum level noted in March and the maximum in August. Another study in a Romanian population [34] also supported the association of seasonal variation with serum 25(OH)D level (highest in September and lowest in March) regardless of study subgroups. It has been demonstrated that vitamin D deficiency impairs bone mineralization and increases bone turnover, thus accelerating bone loss [35,36]. Several studies have also reported that humans in colder regions showed low cortical thickness and bone mineral density, which result in accelerated bone loss with aging [37-39]. The dynamic change of serum 25(OH)D levels may thus have an effect on the bone mass and bone architecture and play a key role in the seasonal variation in OP.

### Limitations

Nevertheless, this study has several limitations that need to be acknowledged. First, Google Trends data did not measure the prevalence, but rather contained the RSV that might be influenced by several confounders. Second, the presence of already known facts and consensus could affect people’s preference when searching the related term of interest. Furthermore, the influence of politics and media was evident in the trends of search volumes, which may result in sampling bias.

Despite these limitations, our study also has several strengths. The study included a large and exhaustive amount of data with a long time span, thus making the results more representative and reliable as compared with a cross-sectional study. Furthermore, the findings of this study are helpful for public health officials to facilitate aid and optimize positive health outcomes by providing resources at the best time for intervention, especially when a majority of people with health-related information needs concerning OP are engaged in the process of information seeking.

### Conclusions

Overall, this study revealed a slow global increase of internet search for “osteoporosis” in recent years, and also showed a significant seasonal variation in global RSV for “osteoporosis.” In addition, the presence of seasonal patterns in RSV for “osteoporosis” was found in 6 English-speaking countries. Public relative search rising topics regarding “osteoporosis” indicated the major public concerns about this disease. This study also provided evidence about the search interest of public and dynamic trends in OP through an internet search, which could provide an initial contact point for patients experiencing symptoms, and may potentially be used to expedite necessary medical evaluation. In addition, as compared with a traditional epidemiological study, web-based data could be used as a supplement to the traditional surveillance data for the early control and prevention of this disease.

## Abbreviations

25(OH)D: 25-hydroxy vitamin D
FRAX: fracture risk assessment tool
OP: osteoporosis
RSV: relative search volume
